# Redirection of metabolic flux in *Shewanella oneidensis* MR-1 by CRISPRi and modular design for 5-aminolevulinic acid production

**DOI:** 10.1186/s40643-021-00366-6

**Published:** 2021-02-07

**Authors:** Ying-Chen Yi, I-Son Ng

**Affiliations:** Department of Chemical Engineering, National Cheng Kung University, Tainan, 70101 Taiwan

**Keywords:** *Shewanella oneidensis* MR-1, CRISPRi, 5-Aminolevulinic acid, Metabolic flux, Heme synthesis, C4 pathway, C5 pathway

## Abstract

Programming non-canonical organisms is more attractive due to the prospect of high-value chemical production. Among all, *Shewanella oneidensis* MR-1 possesses outstanding heme synthesis ability and is well-known for electron transfer, thus has high potential in microbial fuel cell and bioremediation. However, heme, as the final product of C4 and C5 pathways, is regulated by heme cluster for the high-value 5-aminolevulinic acid (ALA) for cancer photodynamic therapy, which has never been explored in MR-1. Herein, the heme metabolism in MR-1 was firstly optimized for ALA production. We applied CRISPR interference (CRISPRi) targeted on the genes to fine-tune carbon flux in TCA cycle and redirected the carbon out-flux from heme, leading to a significant change in the amino acid profiles, while downregulation of the essential *hem*B showed a 2-fold increasing ALA production via the C5 pathway. In contrast, the modular design including of glucokinase, GroELS chaperone, and ALA synthase from *Rhodobacter capsulatus* enhanced ALA production markedly in the C4 pathway. By integrating gene cluster under dual T7 promoters, we obtained a new strain M::TRG, which significantly improved ALA production by 145-fold. We rewired the metabolic flux of MR-1 through this modular design and successfully produced the high-value ALA compound at the first time.

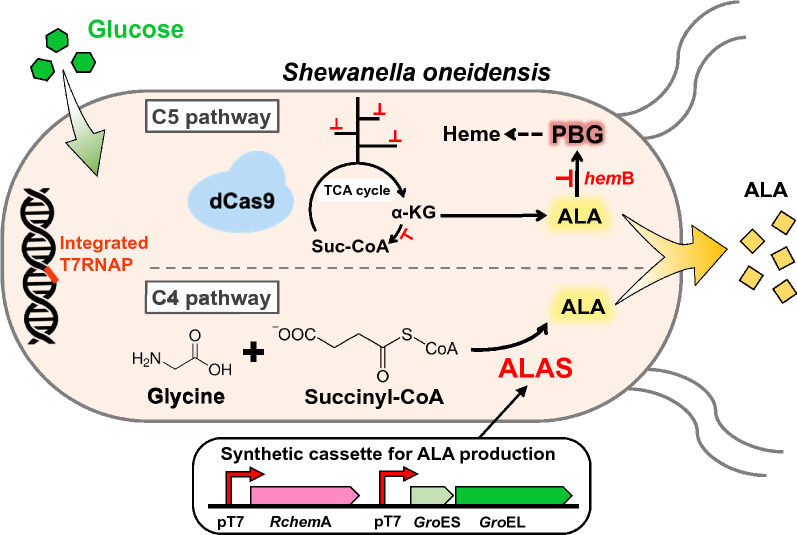

## Introduction

*Shewanella* species are famous for the dissimilatory metabolism of manganese and iron oxides (Ng et al. 2014). *Shewanella* is also capable of electron transfer electron in Mtr pathway which has been widely applied in microbe fuel cells (MFC), remediation in wastewater, removal of organic compounds and azo-dyes and biofabrication of nanoparticles (Wu and Ng 2017; Fredrickson et al. 2008; Hirose et al. 2019; Huang et al. 2019). Recently, MR-1 is feasible in genetic manipulation, since it has a fully annotated genome database (Heidelberg et al. 2002). Genetic MR-1 has mostly been applied to produce acetoin, butanol, bioelectricity and nanoparticles by utilizing different carbohydrates (Choi et al. 2014; Nakagawa et al. 2015; Li et al. 2017). On the other hand, Li and his colleagues exploited a module for NADH regeneration in MR-1 to enhance the extracellular electron transfer (EET) and obtained a 3-folds increment of maximum power output (Li et al. 2018a, b). Moreover, Ng and Yi have explored the different promoters to turn on the Mtr genes in MR-1 (Ng et al. 2018). Studies of MR-1 are from bioelectricity, redistribute central carbon utilization, and biosynthesis of valuable chemicals through genetic and metabolic regulation in the recent years.

Dysregulating and optimizing metabolic mechanisms toward a targeted biosynthesis product is a prevailing and efficient strategy to accelerate the accumulation of bioproducts in microorganisms (Nielsen and Keasling 2016). The development of metabolic engineering relies on synthetic biological tools, while the manipulation of metabolic pathways is usually performed by blocking the branch pathways or repressing the feedback inhibitors to achieve a higher production of the desired products (Noh et al. 2017a). However, metabolic interruption has a physiological impact on bacteria and decreases cellular growth and robustness. Consequently, the partial downregulation of transcriptional factors by CRISPR interference (CRISPRi) is a compelling tool for cellular metabolic engineering (Qi et al. 2013; Ting and Ng 2020). The drawbacks of essential metabolites or the bypassing of the accumulated pathways can be also mitigated by engineered other relevant pathways.

The CRISPRi system is composed of a catalytically deactivated Cas9 protein (dCas9) and a single-guide RNA (sgRNA) (Larson et al. 2013). The dCas9 retains the ability to bind with DNA with the assisted sgRNA containing a target-specific complementary region. The CRISPRi system was developed to regulate the expression of specific genes in several model microorganisms, including *Escherichia coli* (Tian et al. 2019), *Chlamydomonas reinhardtii* (Kao and Ng 2017) and *Corynebacterium glutamicum* (Yoon and Woo 2018) or more. For MR-1, CRISPRi has applied to reduce its EET ability by repressing gene expression in the Mtr pathway (Cao et al. 2017), while a new strategy of tuning electron balance using a CRISPR-ddAsCpf1-based system allowed for the redistribution of the electron flux to enhance EET capacity (Li et al. 2020). Although the feasibility and advantages of the CRISPRi used for MR-1 on improving EET efficiency has been demonstrated, it is rarely used for chemical production.

The C4 and C5 pathways are two indispensable approaches for 5-aminolevulinic acid (ALA) production, which is a non-proteinogenic five-carbon amino acid, as well as an important intermediate in tetrapyrrole synthesis. It has been approved by the FDA for the photodynamic therapy of cancers. MR-1 is remarkable with versatility of over 42 *c*-type cytochromes, which are also predicted to be multi-heme and beneficial to oxidoreduction (Meyer et al. 2004). Due to the outstanding ability of intracellular heme accumulation in cell, MR-1 is considered to have strong pathway for heme synthesis, which leads to higher ALA production. Therefore, we aim to improve the accumulation of ALA in the heme synthesis pathway in MR-1 via C4 and C5 pathways. Firstly, the carbon flux was redirected into the TCA cycle by downregulating the branch pathways using the CRISPRi system. The flux on the upstream of C5 pathway and heme biosynthesis was modulated to increase α-ketoglutarate and _L_-glutamate accumulation. Then, *hemB* expression was fine-tuned by CRISPRi to balance cell survival and prevent ALA from converting into porphobilinogen (PBG). Finally, ALA synthase (ALAS) was co-expressed with the chaperone GroELS, allowing MR-1 to synthesize ALA via C4 pathway. The production of ALA using integrative modular genetic elements in M::TRG was also demonstrated.

## Results and discussion

### Regulation of carbon flux in TCA cycle by CRISPRi

**Fig. 1:**
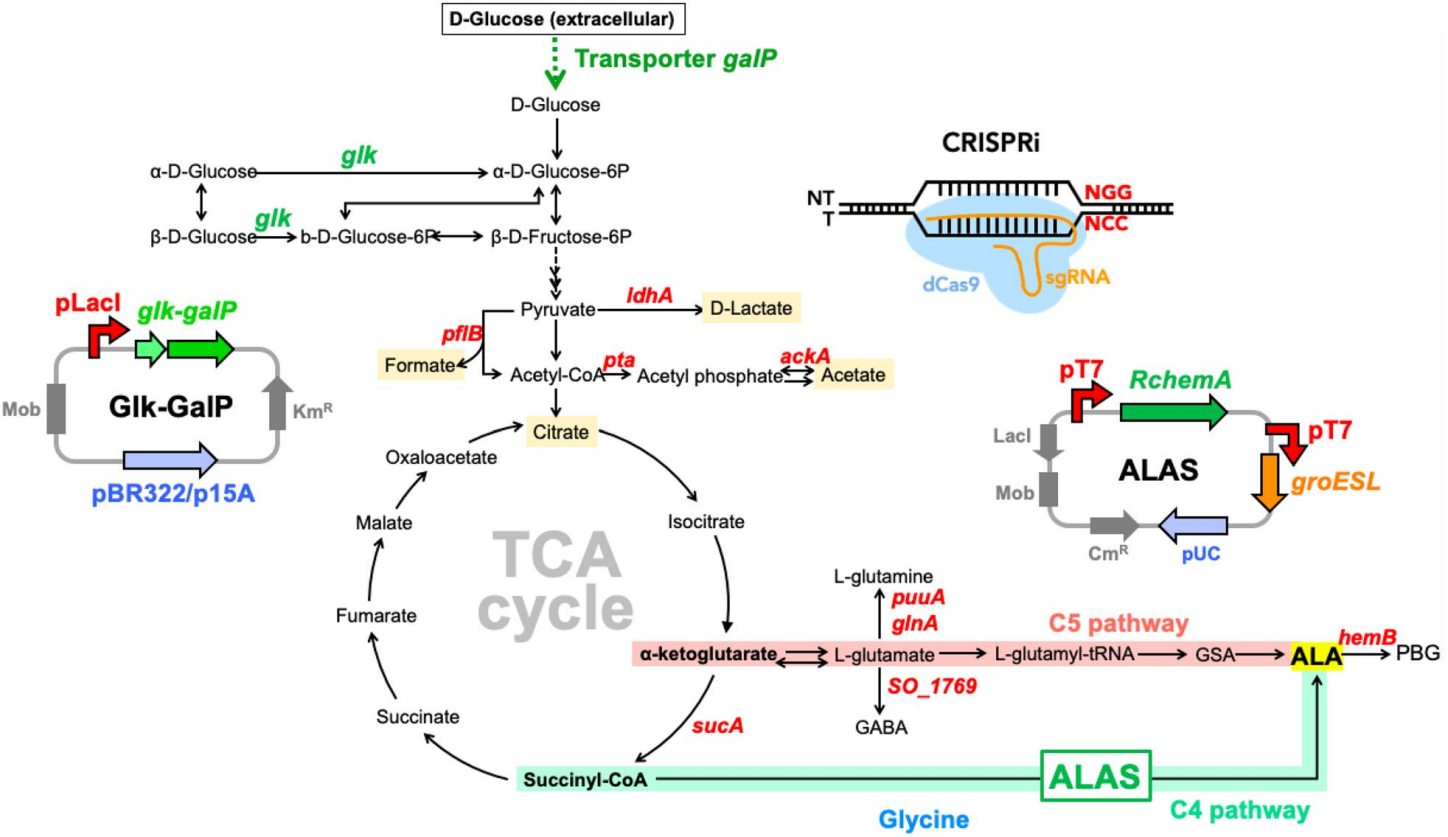
Metabolic pathway and schematic diagram of 5-ALA synthesis from glucose to TCA cycle. Genes shown in red were downregulated using the CRISPRi system, while metabolites represented in yellow were analyzed by HPLC. Two important plasmids for expression of RchemA with GroESL and Glk-GalP were harboring in the genetic MR-1

**Fig. 2:**
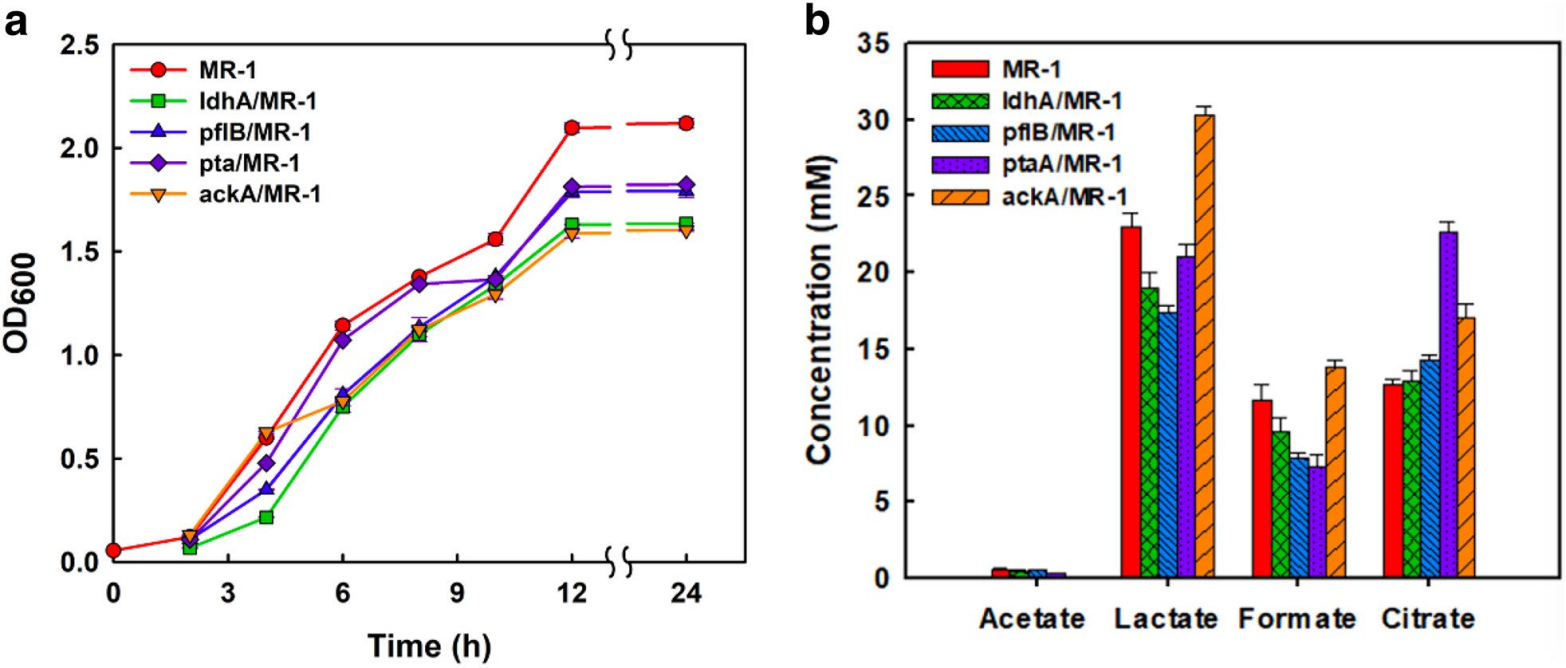
Characterization of *Shewanella* strains via single downregulation of different genes. **a** Growth curve compared to wild-type MR-1 in LB. **b** Metabolites were analyzed by HPLC at 12 h. The genes repressed in MR-1 are indicated

Metabolic pathway and schematic diagram of ALA synthesis from glucose to TCA cycle is shown in Fig. 1. Genes in red were downregulated using the CRISPRi system and to reduce the carbon flux bypassing, where the dCas9 mediated with *ldh*A for lactate, *pfl*B for formate, and *pta* or *ack*A for acetate, respectively. We found that all 4 strains under the control of CRISPRi grew slowly, and biomass obtained after 12 h was lower than that of wild type (Fig. 2a). Afterwards, four major metabolic compounds which including acetate, lactate, formate, and citrate were analyzed. As shown in Fig. 2a, b little amount of acetate was accumulated when culturing MR-1 under aerobic condition. Lactate accumulation in strains with CRISPRi design on *ldh*A, *pfl*B and *pta* were less, while that on *ack*A would be increased to 30 mM, indicating the metabolic flux turned into lactate due to the strongest regulation by *ack*A. However, lactate was not significantly down-regulated in CRISPRi on *ldh*A, which was caused by the low enzyme activity of LdhA, and less inhibition under aerobic condition (Kasai et al. 2019). Thus, it has no effect on citrate accumulation as a downstream metabolite (Fig. 2b).

With *pflB* downregulation, the formate and lactate were reduced by 10% to 15% compared with wild type (Fig. 2b). However, it did not increase citrate accumulation in the TCA cycle. Actually, formate is an important energy source which is essential to improve the internal redox balance during the aerobic or anaerobic respiration of MR-1 (Song et al. 2013). From our result, it implied that down-regulation of *pflB* did not affect the flux from pyruvate to citrate. This was consistent with previous study, which reported a lower pyruvate consumption rate of MR-1 after deletion of the *pflB* gene (Pinchuk et al. 2011), thus was not beneficial to citrate accumulation in the TCA cycle.

Citrate was associated with the highest carbon flux into TCA cycle in CRISPRi on *pta* and *ack*A strains (Fig. 2b). When *pta* was downregulated, the amount of acetate, lactate and formate was reduced. The *pta*/*ack*A pathway was responsible for ATP generation in the TCA cycle and resulting in different cell growth behavior (Hunt et al. 2010). MR-1 was unable to survive after *pta* or *ackA* deletion under anaerobic conditions with glucose or lactate as a carbon source. In this study, only a slight decrease of biomass was associated with dCas9 interference in the aerobic condition with LB medium (Fig. 2a). Moreover, *ackA* was more critical for acetate production than *pta*, that caused carbon flux to bypass into formate, lactate, and other pathways. On the other hand, *pta* transformed the acetyl-CoA to acetyl-phosphate without ATP/ADP reaction and reserved more energy. While down-regulation of *ack*A would reduce the formation of ATP and triggered more metabolites bypassing instead of entering to TCA cycle. Thus, downregulating *pta* was the most effective to redirect the carbon flux into citrate accumulation without other metabolites bypassing in the cell.

### Centralizing the heme synthesis pathway to increase ALA production

**Fig. 3:**
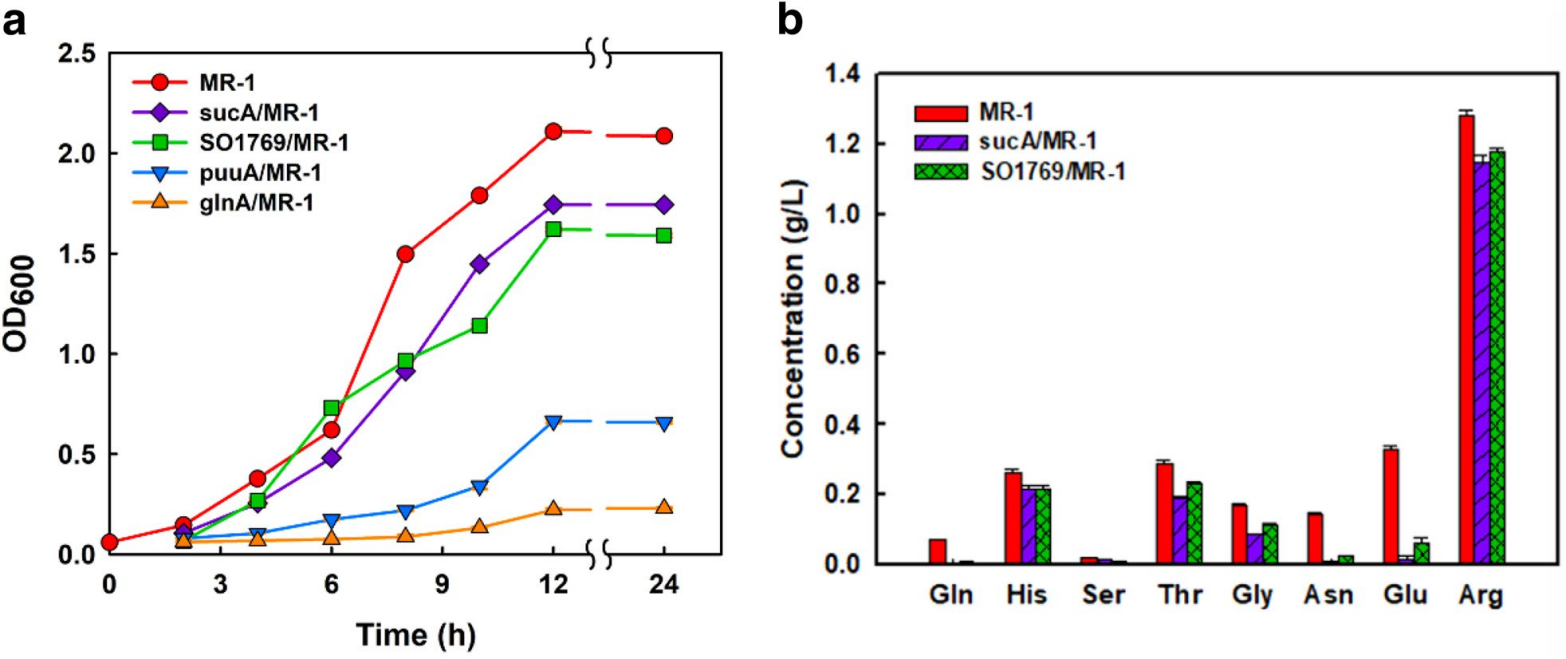
Effect of the interference of genes closely associated with the heme synthesis pathway on *Shewanella* strains. **a** Growth curve of interfered strains compared to that of wild-type MR-1 in LB medium. **b** Analysis of important amino acids by HPLC at 12 h. The genes repressed in MR-1 are indicated. *Gln* glutamine, *His* histidine, *Ser* serine, *Thr* threonine, *Gly* glycine, *Asn* asparagine, *Glu* glutamate, *Arg* arginine

To further enhance ALA production through heme synthesis, genes related to α-ketoglutarate (α-KG) and l-glutamate accumulation were down-regulated (Fig. 1). The *suc*A gene in the TCA cycle was inhibited by dCas9 to increase the accumulation of α-KG, while the amount of l-glutamate was expected to be increased by depressing *gln*A, *puu*A, and *SO_1769*. However, MR-1 was difficult to survive after the downregulation of *gln*A and *puu*A, while strains with *suc*A and *SO_1769* repression reached OD_600_ at 1.5 after 12 h (Fig. 3a). The l-glutamate on the heme synthesis pathway was a precursor for other amino acid synthesis, which was important to cell survival. Down-regulation of *puu*A or *gln*A impaired the cell growth in MR-1 (Fig. 3a). The *gln*A and *puu*A genes were responsible for l-glutamine synthesis which were critical for cell growth due to the catalysis of ATP-dependent γ-glutamylation of putrescine. PuuA was a key enzyme in the synthesis of putrescine, that played an important role in cell proliferation (Kurihara et al. 2008). In addition, the downregulation of *gln*A affected the production of l-arginine from l-glutamate; thus, the regulation and balance of l-glutamine, l-arginine, and putrescine was fundamental for cell growth and activity (Noh et al. 2017b).

Although cell growth was not harmed when *SO_1769* and *suc*A were downregulated, there was no improvement on the ALA biosynthesis by CRISPRi targeting the C5 pathway. Amino acids were quantified using HPLC analysis, as shown in Fig. 3b. The levels of the major amino acids, namely serine, histidine, glutamine, arginine, glutamic acid, asparagine, glycine, and threonine, were found to decrease in the knockdown strains. The cell growth was slightly decreased with the inhibition on *SO_1769* and *suc*A; however, the ALA titer was not improved with the interferences (Fig. 3a). The knock-down of *suc*A might impair the TCA cycle due to the insufficient production of energy and building block, resulting in lower biomass and ALA production (Noh et al. 2017a). According to the NCBI database, *SO_1769* is annotated as a glutamate decarboxylase and putative pyridoxal-dependent aspartate 1-decarboxylase. Therefore, the interference of *SO_1769* would affect the metabolism of alanine and aspartate synthesis and lower the amino acid content, while not being lethal to the cell (Fig. 3b). In addition, *suc*A is encoded as an α-oxoglutarate dehydrogenase, which is an NADH-dependent enzyme and may alter the intracellular electron transfer and energy balance in MR-1. Consequently, enhancing glutamate and ALA accumulation by blocking genes in the heme synthesis pathway is not a promising strategy.

### Downregulation of *hem*B genes on multiple targeting sites by CRISPRi

**Fig. 4:**
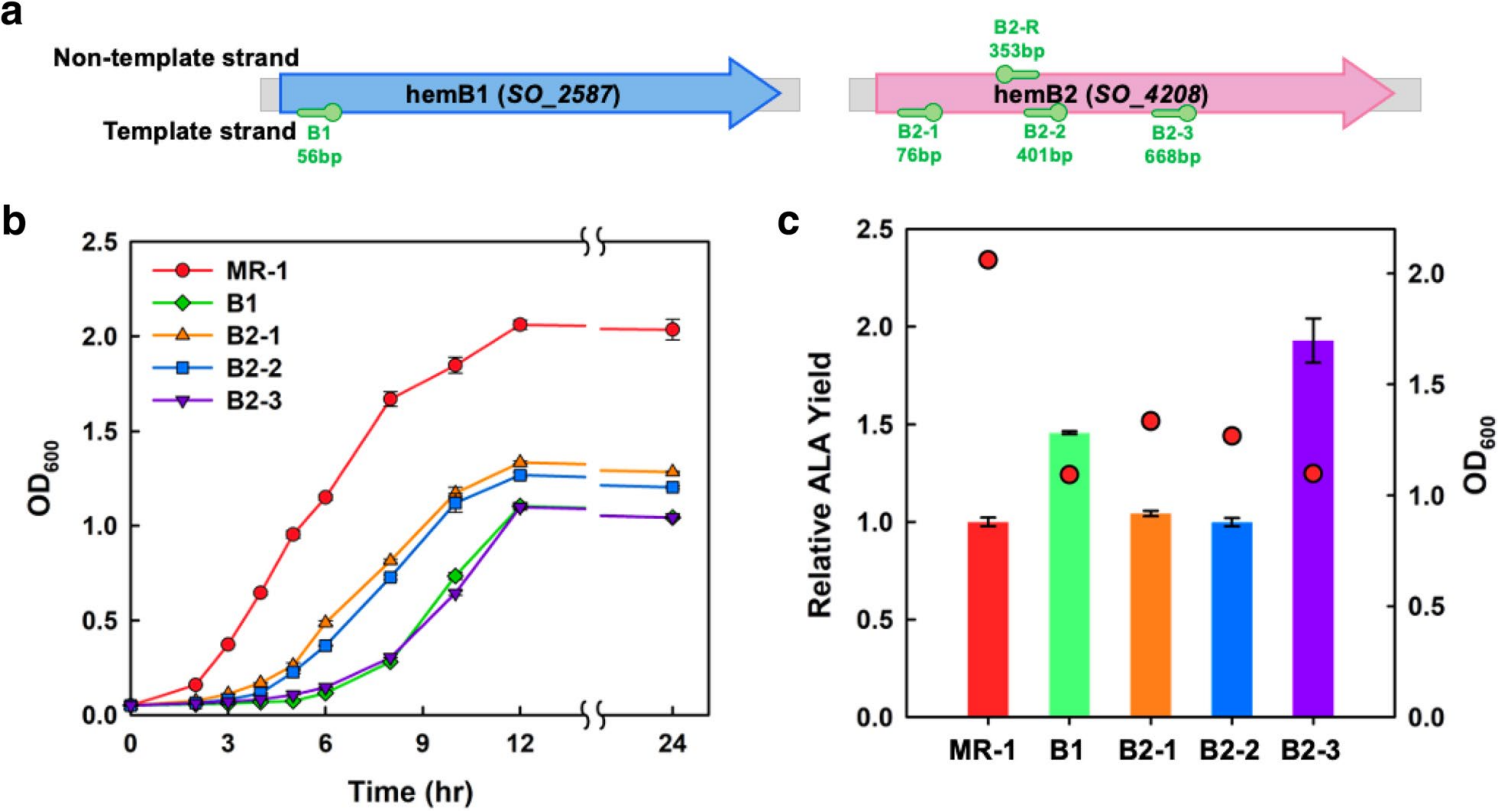
Characterization of different targeting sites on *hemB* genes in MR-1. **a** Several targeting sites for dCas9 were designed on *hem*B2 (*SO_4208*). The growth curve (**b**) and relative ALA yield (**c**) were analyzed from the strains in LB medium at 12 h. The bar in **c** shows the relative ALA yield compared with that in wild-type MR-1. Red dots indicate the OD_600_ of cell

As shown in Fig. 1, the accumulation of ALA could be improved by down-regulating the *hem*B gene, which encoded 5-aminolevulinic acid dehydratase (ALAD) in both C5 and C4 pathways. Five targeting sites for dCas9 on two *hem*B genes (i.e., *SO_2587* as *hem*B1 and *SO_4208* as *hem*B2) were selected to fine-tune the genes expression and cell growth of MR-1 strains B1, B2-1, B2-2, B2-3, and B2-R (Fig. 4a). The cell growth differed from various dCas9 targeting sites on the *hem*B genes (Fig. 4b). Initially, MR-1 could not survive when dCas9 targeting on the non-template strand of *hem*B2 (i.e., B2-R). All the CRISPRi strains grew slowly than the wild type. As a result, the biomass of wild type was the highest at 12 h. For CRISPRi strains, the ranking of cell growth in terms of targeting sites was B2-1 > B2-2 > B1 and equal to B2-3 (Fig. 4b). Thus, ALA production in the strains B2-1 and B2-2 was increased due to the downregulation of *hem*B2 (Fig. 4c). Finally, the HemB activity was fine-tuned, with an optimal expression level of *hem*B2 resulting in a 2-folds increase in ALA production.

The regulation of the C5 pathway significantly affected cell growth and metabolism due to it was being the upstream pathway of heme, porphyrin, and vitamin B_12_. Moreover, the downregulation of *hem*B reduced the metabolic flux of ALA to downstream metabolites, and, therefore, impaired the negative feedback inhibition of the ALA synthesis pathway (Zhang et al. 2015), which was advantageous to ALA accumulation. However, the cell growth would be attenuated when *hem*B was knock-down and resulted in low biomass, which is a critical shortage for chemical production in industry.

### Production of ALA in C4 pathway with CRISPRi system

**Fig. 5:**
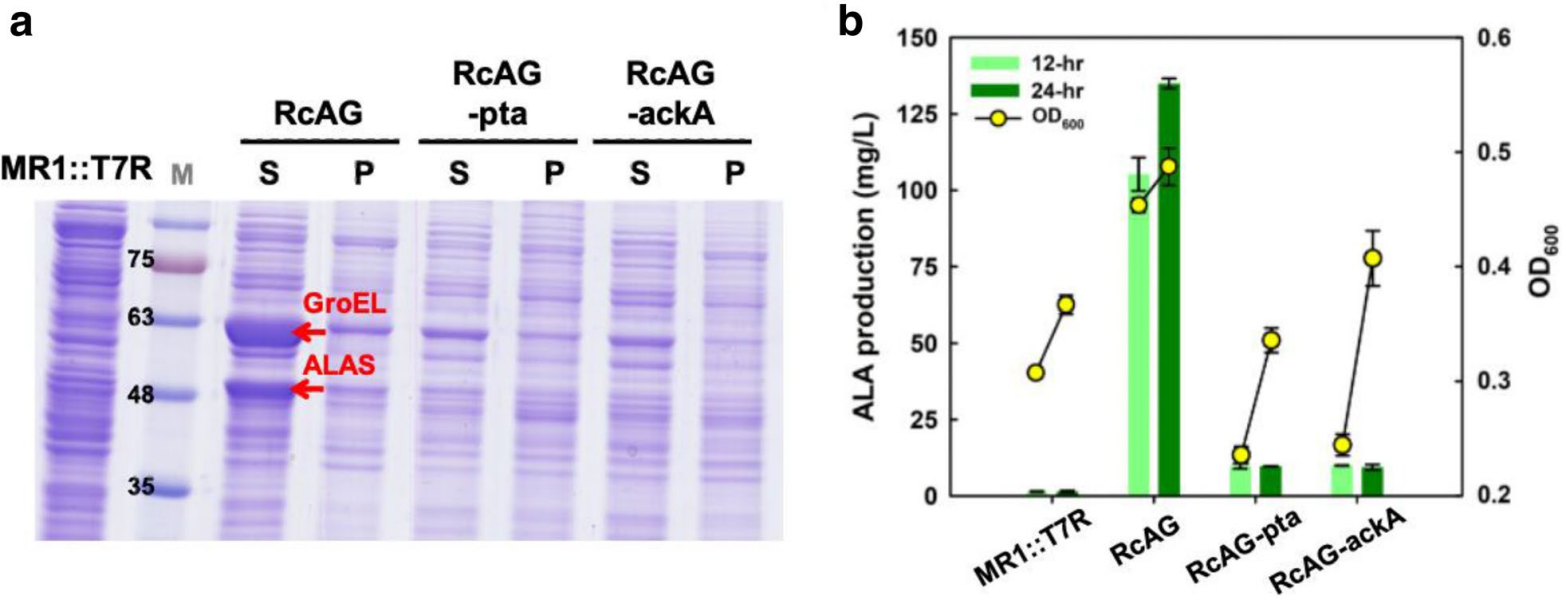
Co-expression of ALAS and GroELS in a single plasmid in MR1::T7R with *pta* and *ack*A downregulation in MM9 media. **a** Protein expression was observed by SDS-PAGE at 12 h. The arrows indicate ALAS and GroEL at sizes of approximately 48 kDa and 63 kDa, respectively. *S* soluble protein, *P* insoluble protein, *M* protein ladder marker. **b** ALA production and biomass in terms by OD_600_were analyzed at 12 h and 24 h, respectively

The C4 pathway using ALAS from *R. capsulatus* was used in MR1::T7R, which contained T7 RNA polymerase (T7RNAP) in the chromosome (Yi and Ng 2020). The soluble form of ALAS has been increased in the presence of GroELS to achieve a higher ALA yield in *E. coli* (Yu et al. 2020). Moreover, GroELS assisted the folding of different enzymes have been demonstrated (Effendi et al. 2020). Therefore, the strain RcA-EcG was used to co-express ALAS with GroELS under the T7 promoter and was compared with the *pta* and *ack*A-interfered strains. Protein analysis showed that ALAS and GroEL were only overexpressed in MR1::T7R without the CRISPRi system, while the expression levels dramatically decreased in the *pta* and *ack*A-interfered strains (Fig. 5a). ALA production was found to be directly related with ALAS expression (Fig. 5b). On the other hand, MR-1 was unable to consume glucose as a carbon source, which the growth in MM9 showed a much lower biomass in terms of OD_600_ (Fig. 5b) than that in LB medium (Fig. 4c). Moreover, no marked ALAS or GroEL expression was observed in the dual-plasmid strains (i.e., RcA-EcG with pSM-dCas9-pta or pSM-dCas9-ackA). The biomass was found critically low at 12 h and increased linearly at 24 h. As the result, there was no significant improvement on ALA production in RcAG-pta and RcAG-ackA due to the low expression of ALAS (Fig. 5b). Compared to starting strain and CRISPRi derivatives, the significant improvement of cell growth, with an OD_600_ of 0.5 and ALA production of 134 mg/L was achieved by the RcA-EcG strain. As we found, ALA production was highly relevant with ALAS expression (Fig. 5a, b). Even the feasibility of protein expression by the dual-plasmids in MR-1 which cultured in Luria–Bertan (LB) medium has been verified (Cao et al. 2019), the stress of different genes and medium condition would alter the results. LB is a rich medium for most bacteria but is not favored to produce ALA (Yu et al. 2020). Due to MR-1 is difficult to grow in minimal medium, expressing of dual plasmids for ALA production is still challenge.

### Engineered glucose-utilizing strains for ALA production

**Fig. 6:**
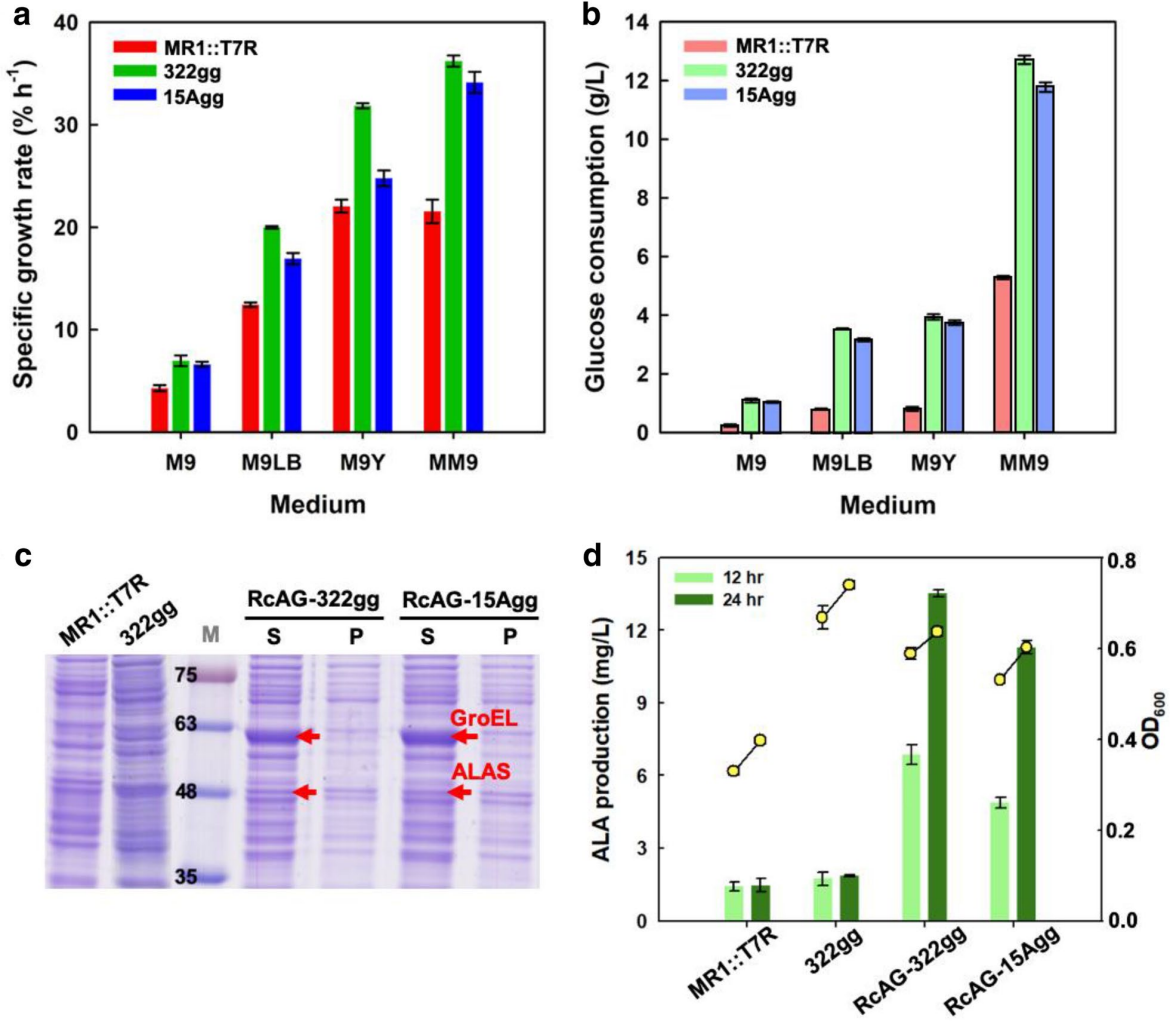
Characterization of glucose-consuming strains in different minimal media. MR-1 cells with medium and low copy numbers for *glk-gal*P were compared with the wild-type strain, and the specific growth rates (**a**) and glucose consumption (**b**) were analyzed. ALAS and GroELS were co-expressed with *glk-gal*P as two plasmids. **c** Protein expression was observed by SDS-PAGE at 12 h, which ALAS and GroEL at sizes of approximately 48 kDa and 63 kDa, respectively. *S* soluble protein, *P* insoluble protein, *M* protein ladder marker. **d** ALA production and biomass in terms by OD_600_ were analyzed at 12 h and 24 h

Since glucose is not only the carbon source in the medium but also serves as an inhibitor of ALAD to increase ALA accumulation (Howard et al. 2012), that making MR-1 consume glucose is necessary in this study. The transporter *gal*P and glucokinase *glk* from *E. coli* were expressed in MR-1 to improve the growth in minimal medium which was used glucose as carbon source. All the strains are shown in Table 1. M9, M9LB, M9Y, and MM9 minimal media with different components were used for cell growth of different MR-1 strains (Additional file 1: Fig. S1). We further analyzed the effect on the specific growth rate (Fig. 6a) and glucose consumption (Fig. 6b). A higher biomass was obtained when using yeast extract in the M9Y and MM9 media. The expression of *glk*-*gal*P markedly enhanced the specific growth rates and glucose consumption in the four media. The highest specific growth rate and glucose consumption were observed in strain 322gg due to the high plasmid copy number (PCN) of pYCI-gg (i.e., 77.1). Contrastingly, a lower specific growth rate was observed for strain 15Agg, with a PCN of only 7.2 (Table 2).

**Table 1:** Strains used in this study

Strains	Description	Source
*E. coli* DH5α	Cloning strain, *F*^*–*^*endA1 glnV44 thi-1 recA1 relA1 gyrA96 deoR nupG purB20 φ80dlacZΔM15 Δ(lacZYA-argF)U169, hsdR17(r*_*K*_^*–*^*m*_*K*_^+^), λ^–^	Lab stock
*E. coli* WM3064	Donor strain for conjugation*, thrB1004 pro thi rpsL hsdS lacZΔM15 RP4-1360 Δ(araBAD)567 ΔdapA1341::[erm pir]*	Lab stock
*S. oneidensis* MR-1	Wild type strain	Lab stock
MR1::T7R	Integration of T7 RNA polymerase onto wild type *S. oneidensis* MR-1 genome	Yi and Ng (2020)
RcAG	MR1::T7R harboring plasmid of RcA-EcG	This study
RcAG-pta	MR1::T7R harboring pSUKM-RcA-EcG and pSM-dCas9-pta	This study
RcAG-ackA	MR1::T7R harboring pSUKM-RcA-EcG and pSM-dCas9-ackA	This study
15Agg	MR1::T7R harboring pYCI-15A-gg	
322gg	MR1::T7R harboring pYCI-gg	This study
RcAG-322gg	MR1::T7R harboring pSUM-RcA-EcG and pYCI-322-gg	This study
RcAG-15Agg	MR1::T7R harboring pSUM-RcA-EcG and pYCI-15A-gg	This study
M::TRG	MR1::T7R with integration of *Rchem*A and *Ecgro*ELS under T7 promoter	This study

**Table 2:** Plasmid copy number of different strains

Strain	Gene of interest	PCN
15Agg	*glk*	7.2
322gg	*glk*	77.1
RcAG	*RchemA*	118.1
RcAG-15Agg RcAG-15Agg	*glk*	14.9
	*RchemA*	292.4
RcAG-322gg RcAG-322gg	*glk*	31.8
	*RchemA*	127.5

Both the RcAG-322gg and RcAG-15gg strains harboring the dual plasmids were cultured in MM9 medium as the optimal condition for ALA production. As aforementioned, the expression of ALAS and GroEL were abundant in RcAG (Fig. 5a). However, only a trace amount of ALAS was observed in the glucose-consuming strains (i.e., RcAG-322gg and RcAG-15Agg) (Fig. 6c). As shown in Table 2, the PCN of pSUM-RcG-EcA increased to 127.5 and 292.4 with co-existence of pYCI-gg and pYCI-15A-gg, respectively. However, the amount of ALA production was critical low when using dual-plasmid system. Therefore, the transcription level of proteins in the dual-plasmid system might be thwarted in MR1::T7R. Despite the higher biomass obtained from strains with *glk-galP* in MR1::T7R, ALA content was similar to that of WT strain, as shown in Fig. 6d, indicated by the reduced expression of ALAS in the dual-plasmid strains. Consequently, ALA production in the RcAG-322gg and RcAG-15Agg were within 13.2 mg/L.

MR-1 was unable to uptake and phosphorylate glucose due to a frameshift mutation in the glucose/galactose transporter gene and lack of glucokinase (Nakagawa et al. 2015; Li et al. 2017). The transporter *gal*P and glucokinase *glk* from *E. coli* enabled MR-1 to survive with glucose as the sole carbon source. Interestingly, the cell was able to reach higher specific growth rate and more glucose consumption with more yeast extract (Fig. 6a, b). The genus *Shewanella* has great flexibility and proliferation in diverse environments; such characteristics allow it to utilize glucose as a sole carbon source during glucose exposure (Howard et al. 2012). The growth rate of MR-1 with dual plasmids was improved by introducing *glk-gal*P, while ALA accumulation was restricted due to ALAS expression. As a result, the highest ALA content (134 mg/L) was achieved by expressing ALAS and GroELS in a strain with a single plasmid (i.e., RcAG). The expression of heterologous proteins in two plasmids was required further investigation.

### Fine-tuned ALA production using modular design in an integrative strain

**Fig. 7:**
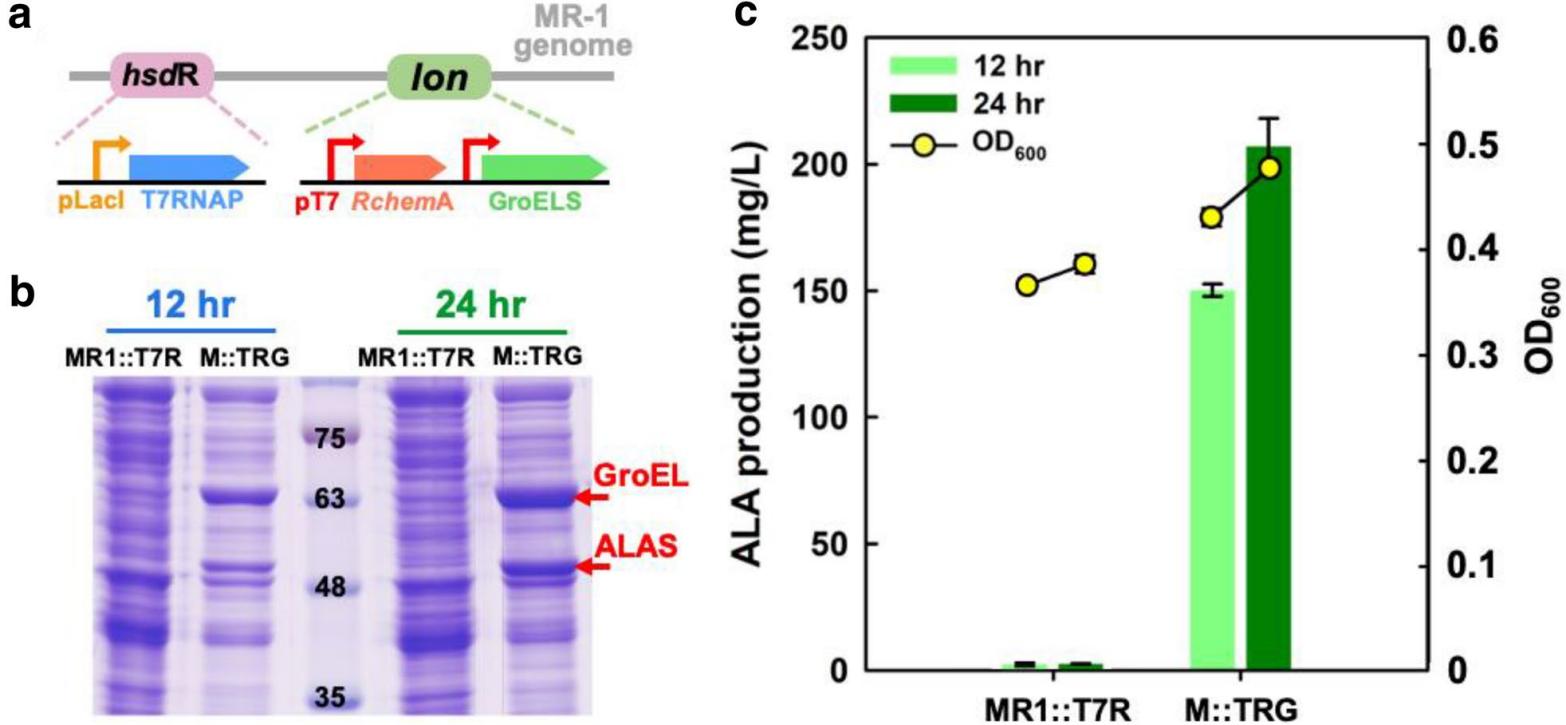
Analysis of integrated strains in MM9 medium. **a** Schematic diagram of MR-1 integration with T7 RNA polymerase (T7RNAP) under pLacI on the *hsd*R site and *Rchem*A and GroELS under dual T7 promoters on the *lon* gene. **b** SDS-PAGE analysis of MR1::T7R and M::TRG. The arrows indicate the ALAS and GroEL at sizes of approximate 48 kDa and 63 kDa, respectively. **c** ALA production and biomass in terms by OD_600_ were analyzed at 12 h and 24 h

High levels of heterologous protein expression can be obtained in the plasmid, but it may encounter the genetic instability. Therefore, antibiotics are necessary as a selective pressure to induce a high copy number. Such disadvantages can be overcome using gene integration to the chromosome in microorganisms. Alleviating cell stress from plasmid duplication for resistance to antibiotic by integration strategy may enable the cell to fine-tune the expression and metabolism for its growth, which further increase ALA accumulation in the cell. Therefore, *Rchem*A and *gro*ELS under dual T7 promoters were integrated into the MR1::T7R strain with the homologous region of the *lon* gene to obtain the strain M::TRG (Fig. 7a). The protein expression of ALAS and GroEL was observed using SDS-PAGE after 12 h and 24 h of cultivation in MM9 (Fig. 7b). The amount of both ALAS and GroEL, as well as ALA production, was found to increase from 12 to 24 h (Fig. 7c). At 12 h, the amount of ALAS in M::TRG reached 150.3 mg/L (Fig. 7c), while RcAG obtained 105 mg/L (Fig. 5b). These results demonstrated the advantages of the integration strategy over the use of plasmid-based system for chemical production in microorganisms. Lastly, ALA production of 207 mg/L was much improved by integrating *Rchem*A and *gro*ELS to the chromosome.

From the beginning, a step-by-step process comprising the CRISPRi system was used to explore the metabolic flux in MR-1. However, only 9.9 mg/L and 9.4 mg/L of ALA were produced by the RcAG-pta and RcAG-ackA strains with CRISPRi. No positive effect was observed on the dual-plasmid system. A summary of ALA production in MR-1 using different approaches via the C4 pathway is provided in Table 3. The amount of ALA was increased from 1.42 mg/L in the parent strain to 207 mg/L in the best strain M::TRG by 145-folds increasement at 24 h.

**Table 3:** Summary of ALA production in comparison with MR1::T7R at 24 h in this study

Strain	ALA titer (mg/L)	Improvement (folds)
MR1::T7R	1.42	1
RcAG-pta	9.9	7.0
RcAG-ackA	9.4	6.6
RcAG-15Agg	11.1	7.8
RcAG-322gg	13.2	9.3
RcAG	134.9	95.0
M::TRG	207.0	145.8

## Conclusion

Microorganisms utilize a variety of metabolic pathways from different carbon sources, to allowing the advantages in their unique functions. The uptake of energy and the regulation of metabolic flux are crucial for the growth and accumulation of metabolites in bacteria. In this study, the metabolic flux of *S. oneidensis* MR-1 was elucidated by applying the CRISPRi system to knock down the branch pathways of glycolysis. The genes of *puu*A and *gln*A are essential for cell growth. The regulation of heme synthesis pathway improved the ALA accumulation through the C5 pathway, while the integration of *Rchem*A and *gro*ELS to the chromosome was the best approach to obtain the highest ALA in MR-1 with T7RNA polymerase. Although ALA production is still low, the redirection of metabolic flux by CRISPRi and different modular designs are explored to reveal the regulation of C4 and C5 pathways in MR-1.

## Materials and methods

### Bacterial strains and culture conditions

All bacterial strains are listed in Table 1. The *E. coli* strains, DH5α and WM3064, were cultivated in Luria–Bertani (LB) medium at 37 °C with shaking at 200 rpm. The wild-type and recombinant MR-1 strains were cultivated in all media at 30 °C with shaking at 150 rpm. For the glucose consumption analysis, M9 minimal medium (M9) and three modified M9 media were used (Additional file 1: Table S1). For ALA production via C4 pathway, the cell culture was conducted in 250-mL Erlenmeyer flasks containing 50 mL minimal medium. A single colony was inoculated into a 15-mL tube containing 3 mL of LB medium. Antibiotics were added at the following concentrations: 50 µg/mL kanamycin or 25 µg/mL chloramphenicol. The antibiotic concentration was halved for the dual-plasmid strains. The cultivation medium of *E. coli* WM3064 was supplemented with 0.3 mM of 2,6-diaminopimelic acid (DAP). For the induction culture of the MR-1 strains, a final concentration of 0.5 mM isopropyl-*d*-1-thiogalactopyranoside (IPTG) was added when the optical density at 600 nm (OD_600_) reached 0.2 during cultivation. All cultures for ALA production with ALAS were supplemented with 3 g/L glycine, 1 g/L succinate, and 30 μM pyridoxal phosphate (PLP) during IPTG induction.

### Plasmid construction and transformation

All plasmids used and constructed in this study are listed in Table S2. Plasmid construction was performed in *E. coli* DH5α. Mobilization gene cluster (mob), including *oriT*, conjugative transfer protein, and TraI were inserted into pdCas9 to obtain pSM-dCas9. All sgRNA for dCas9 targeting were annealed and inserted into pSM-dCas9. The sequences of the designed sgRNAs are listed in Table S3. The *glk* and *gal*P genes were amplified from *E. coli* by PCR and overlap-PCR before their insertion into the pYCI plasmid. The plasmid pYCI-15A-gg was constructed from pYCI-gg by the substitution of p15A origin for pUC origin. The ALAS was used our previous study (Yu et al. 2020), while chaperone GroELS was amplified from *E. coli* and constructed as pSUM-RcA-EcG. The resistance of chloramphenicol (Cm^R^) in pSUM-RcA-EcG was replaced with kanamycin (Km^R^) for dual-plasmid expression using the CRISPRi system. Because the wild-type MR-1 does not possess T7 RNA polymerase (T7RNAP) for the orthogonal T7 system, MR1::T7R was selected as the host for protein expression under T7RNAP/T7 (Yi and Ng 2020). pMobS was constructed from pDS3.0, replacing gentamicin (Gm^R^) with spectinomycin (Spc^R^). The homologous region of *lon* was amplified from MR-1 and overlap-PCR with *Rchem*A and *gro*ELS before being inserted into pMobS to obtain pMobS-lon-RG. The transformation into *E. coli* strains was performed using heat shock, while transformation into MR-1 and MR1::T7R was performed using conjugation.

### Analytical assays

For protein expression, the cells were washed twice and adjusted to an OD_600_ of 4 using deionized water as the sample. The sample was then disrupted using OneShot^®^ (Constant Systems, UK) to obtain soluble and insoluble proteins. SDS-PAGE with 10% separating gel and 4% stacking gel was used to observe protein expression. Proteins were visualized by staining with Coomassie blue R-250 and scanned using Image Scanner (Biolab2000, Taiwan).

The samples for ALA quantification were derivatized with acetylacetone and then analyzed using modified Ehrlich’s reagent (Yu et al. 2020). The relative ALA production in *hemB*-downregulated strains was compared to that in the wild-type strain. The glucose concentration in the medium was determined using dinitrosalicylic acid (DNS) reagent as previously reported (Saqib and Whitney 2011).

To determine the plasmid copy number by qPCR, the bacterial cells were collected by centrifugation, washed twice with deionized water, and then resuspended in deionized water before incubating at 100 °C for 10 min for cell lysis. After centrifugation, the supernatant was subjected to qPCR using EvaGreen qPCR System-ROX I (GeneDireX, Germany) and StepOnePlus™ Real-Time PCR System (Applied Biosystems, CA, USA). The *mtrA* gene was selected as the reference gene for copy number evaluation in MR-1. The PCN was calculated according to the 2^−ΔCt^ method.

### HPLC analysis

To determine the quantity of extracellular metabolites and amino acids, 1 mL of culture broth was harvested. The samples were subjected to high-performance liquid chromatography (HPLC) (Hitachi, Japan) using a UV detector. For the detection of the metabolites, an ICSep COREGEL-87H3 column (Concise Separations, USA) was used with a holding temperature of 70 °C and a detection wavelength of 210 nm. Diluted sulfuric acid (0.008 N) was used for the HPLC mobile phase with a flow rate of 0.4 mL/min. The samples for amino acid analysis needed to be derivatized for signal emission. This was performed by reacting 340 μL of borate buffer 0.05 M (pH 9) and 240 μL of 100% methanol with 6 μL of sample and 12 μL of 200 mM diethyl ethoxymethylenemalonate (DEEMM). The samples were heated at 70 °C for 2 h to allow for the complete degradation of excess DEEMM and derivatization (Xue et al. 2020). A YMC-Triart C18 column (YMC, Japan) was used to analyze the derivatives at 280 nm at a temperature of 35 °C and a flow rate of 1 mL/min. Mobile phase A (25 mM CH_3_COONa) and mobile phase B (methanol) were adjusted for gradient flow, as follows: 0–8 min, 80–70% A; 8–25 min, 70–65% A; 25–60 min, 65–40% A; 60–63 min, 40–80% A; 63–65 min, 80% A.

### Supplementary Information

**Additional file 1**: **Figure S1**. The growth curves of MR-1 strains in different minimal mediums were analyzed for 12-h cultivation. **Table S1.** Composition and ingredients of minimal mediums, **Table S2.** Plasmids used in this study. **Table S3**. sgRNAs designed in this study.

## Data Availability

The authors approved the availability of data and materials for publishing the manuscript.

## Abbreviations

MR-1: *Shewanella oneidensis* MR-1
ALA: 5-Aminolevulinic acid
MFC: Microbe fuel cells
EET: Extracellular electron transfer
CRISPRi: CRISPR interference
sgRNA: Single-guide RNA
PBG: Porphobilinogen
ALAS: 5-Aminolevulinic acid synthase
α-KG: α-Ketoglutarate
ALAD: 5-Aminolevulinic acid dehydratase
LB: Luria–Bertani
DAP: 2,6-Diaminopimelic acid
IPTG: Isopropyl-*d*-1-thiogalactopyranoside
mob: Mobilization cluster
T7RNAP: T7 RNA polymerase
SDS-PAGE: Sodium dodecyl sulfate–polyacrylamide gel electrophoresis
HPLC: High-performance liquid chromatography
DEEMM: Diethyl ethoxymethylenemalonate
