# Supplementary material for: Redirection of metabolic flux in Shewanella oneidensis MR-1 by CRISPRi and modular design for 5-aminolevulinic acid production
Source: Bioresour Bioprocess. 2021 Feb 7;8(1):13. doi: 10.1186/s40643-021-00366-6 (PMC10992681; doi:10.1186/s40643-021-00366-6)
Supplement: Supplementary file 1 — Additional file 1: Figure S1. The growth curves of MR-1 strains in different minimal mediums were analyzed for 12-h cultivation. Table S1. Composition and ingredients of minimal mediums, Table S2. Plasmids used in this study. Table S3. sgRNAs designed in this study. [file 40643_2021_366_MOESM1_ESM.docx]

**Additional file 1**

Redirection of metabolic flux in *Shewanella oneidensis* MR-1 by CRISPRi and modular design for 5-aminolevulinic acid production

Ying-Chen Yi, I-Son Ng^*^

Department of Chemical Engineering, National Cheng Kung University, Tainan 70101, Taiwan

***Correspondence:** Prof. I-Son Ng

**E-mail**: [yswu@mail.ncku.edu.tw](mailto:yswu@mail.ncku.edu.tw)

Tel: +886-62757575-62648; Fax: +886-62344496

ORCID

**I-Son Ng:** 0000-0003-1659-5814

**Ying-Chen Yi:** 0000-0002-2036-2370


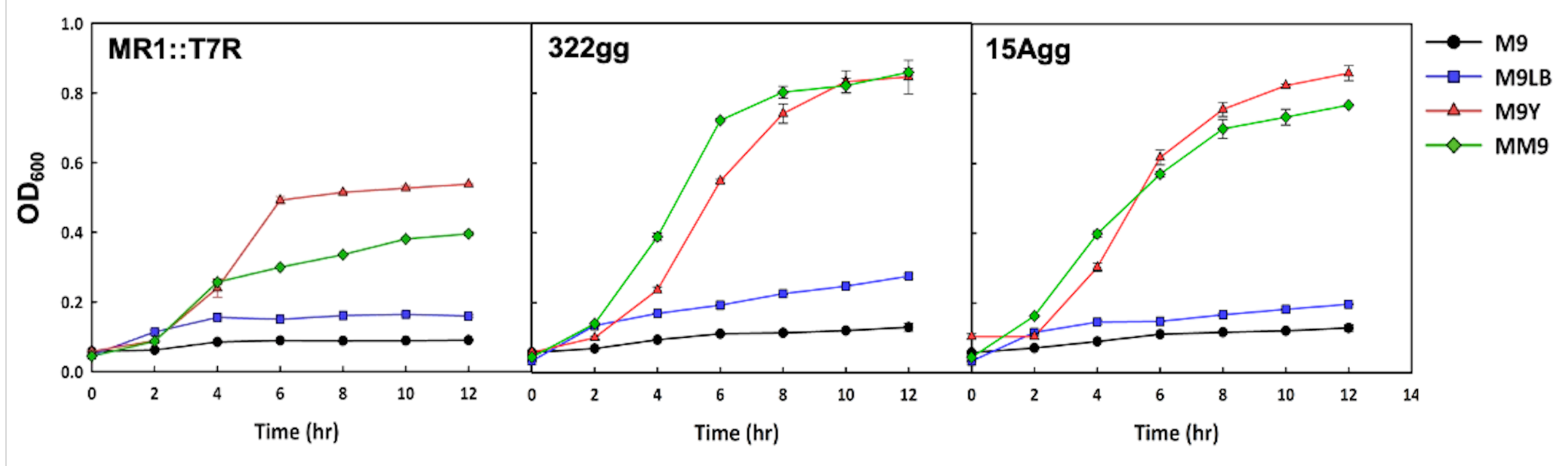


**Figure S1**. The growth curves of MR-1 strains in different minimal mediums were analyzed for 12-h cultivation.

**Table S1.** Composition and ingredients of minimal mediums

| Composition (g/L) | **M9** | **M9LB** | **M9Y** | Composition (g/L) | **MM9** |
| --- | --- | --- | --- | --- | --- |
| Na_2_HPO_4_-12H_2_O | 17.1 | | | Na_2_HPO_4_-12H_2_O | 16 |
| KH_2_PO_4_ | 3.0 | | | KH_2_PO_4_ | 3.0 |
| NaCl | 0.5 | | | (NH_4_)_2_SO_4_ | 16 |
| NH_4_Cl | 0.5 | | |  |  |
| MgSO_4_ | 0.24 | | | MgSO_4_-7H_2_O | 1.0 |
| CaCl_2_ | 0.1 | | | MnSO_4_-H_2_O | 0.01 |
| Glucose | 4.0 | | | Glucose | 20 |
| Yeast Extract | 0 | 0.1 | 2.0 | Yeast Extract | 2.0 |
| Tryptone | 0 | 0.2 | 0 |  |  |

**Table S2.** Plasmids used in this study

| **Plasmid** | Description | Sources |
| --- | --- | --- |
| pdCas9 | 9326 bp, Cm^R^, p15A ori, expresses tracrRNA and dCas9, gRNA scaffold for insertion of target sequence | Addgene #46569 |
| pDS3.0 | Ap^R^, Gm^R^, derived from suicide vector pCVD442 | Lab stock |
| pSM-dCas9 | pdCas9 with mob | This study |
| pSM-dCas9-ldhA | pSM-dCas9 with sgRNA for *ldh*A gene | This study |
| pSM-dCas9-pta | pSM-dCas9 with sgRNA for *pta* gene | This study |
| pSM-dCas9-ackA | pSM-dCas9 with sgRNA for *ack*A gene | This study |
| pSM-dCas9-pflB | pSM-dCas9 with sgRNA for *pfl*B gene | This study |
| pSM-dCas9-SO1769 | pSM-dCas9 with sgRNA for *SO1769* gene | This study |
| pSM-dCas9-sucA | pSM-dCas9 with sgRNA for *suc*A gene | This study |
| pSM-dCas9-glnA | pSM-dCas9 with sgRNA for *gln*A gene | This study |
| pSM-dCas9-puuA | pSM-dCas9 with sgRNA for *puu*A gene | This study |
| pSM-dCas9-hemB1 | pSM-dCas9 with sgRNA for *hem*B*-*1 gene | This study |
| pSM-dCas9-hemB2-1 | pSM-dCas9 with sgRNA for *hem*B*-*2 gene at 76 bp | This study |
| pSM-dCas9-hemB2-2 | pSM-dCas9 with sgRNA for *hem*B*-*2 gene at 401 bp | This study |
| pSM-dCas9-hemB2-3 | pSM-dCas9 with sgRNA for *hem*B*-*2 gene at 668 bp | This study |
| pSM-dCas9-hemB2-R | pSM-dCas9 with sgRNA for *hem*B*-*2 gene at NT strand | This study |
| pYCI-gg | Km^R^, pBR322 ori, mob, pLacI, *glk*, *gal*P | This study |
| pYCI-15A-gg | Km^R^, p15A ori, mob, pLacI, *glk*, *gal*P | This study |
| pSUM-RcA-EcG | Cm^R^, pUC ori, mob, *lac*I repressor, dual pT7, *Rchem*A, *Ecgro*ELS | This study |
| pSUKM-RcA-EcG | Km^R^, pUC ori, mob, *lac*I repressor, dual pT7, *Rchem*A, *Ecgro*ELS | This study |
| pMobS-lon-RG | Spc^R^, R6K ori, mob, lon homologous region, pT7, *Rchem*A, *Ecgro*ELS | This study |

Cm^R^: Chloramphenicol; Km^R^: kanamycin; Spc^R^: spectinomycin

**Table S3**. sgRNAs designed in this study

| **Name** | **Sequence (5’🡺3’)** | **Remark** |
| --- | --- | --- |
| hemB1 | GCAGGCGTCTTAAACGGCGA | hemB gene of SO_2587 |
| hemB2-1 | TGATTTTAGTCGTCGCCTGA | hemB gene of SO_4208 |
| hemB2-2 | CTAGATCCTTTCACGACCCA |  |
| hemB2-3 | GGCTCAGCAGGCAACCTAAA |  |
| hemB2-R | CGGTCATAATGCCAAGTTGC |  |
| pflB | TTTATCCCACAACTGGGTTG |  |
| ackA | GTCTTCTAAGCCAAAACACT |  |
| pta | TTCGGACGAAGTTGTGAAAT |  |
| ldhA | GCAAACGACCTCGAAACCTT |  |
| glnA | TCAGCGTCTACCTGATGAGA |  |
| puuA | CAATCGTCAGAAACTGACTG |  |
| sucA | GTTTGTAGCAAGACACTTTC |  |
| SO_1769 | TGATAGCGGCTTTTCTAGCG |  |
